# New 14-Membered Cyclopeptide Alkaloids from *Zizyphus oxyphylla* Edgew

**DOI:** 10.3390/ijms130911520

**Published:** 2012-09-14

**Authors:** Waqar Ahmad Kaleem, Muhammad Nisar, Mughal Qayum, Muhammad Zia-Ul-Haq, Achyut Adhikari, Vincenzo De Feo

**Affiliations:** 1 Department of Pharmacy, Abdul Wali Khan University Mardan, Anbar Campus 23200, Pakistan; E-Mail: waqar679@yahoo.co.uk; 2 Institute of Chemical Sciences, University of Peshawar, Peshawar 25120, Pakistan; E-Mail: akhund54@gmail.com; 3 Institute of Pharmaceutical Sciences, Kohat University of Science and Technology, Kohat 2600, Pakistan; E-Mail: mu_afridii@yahoo.com; 4 Research Institute of Pharmaceutical Sciences, Department of Pharmacognosy, University of Karachi, Karachi 75270, Pakistan; E-Mail: ahirzia@gmail.com; 5 H.E.J. Research Institute of Chemistry, International Center for Chemical and biological Sciences, University of Karachi, Karachi 75270, Pakistan; E-Mail: achyut.adhikari@iccs.edu; 6 Department of Pharmaceutical and Biomedical Sciences, Universiy of Salerno, Salerno 84100, Italy

**Keywords:** *Zizyphus oxyphylla* Edgew, cyclopeptide alkaloids, antibacterial activity

## Abstract

Two new 14-membered cyclopeptide alkaloids, Oxyphylline B (**4**) and Oxyphylline C (**5**), along with three known 13-membered cyclopeptide alkaloids, were isolated from stem and roots of *Zizyphus oxyphylla* Edgew. The compounds were tested for antibacterial activity. Oxyphylline B (**4**) showed comparatively better antibacterial activities against *Escherichia coli* (MIC, 5 μg/mL) than other compounds. This compound also exhibited weak antimicrobial activities against *Staphylococcus aureus* (MIC, 25 μg/mL), *Pseudomonas aeruginosa* (MIC, 50 μg/mL) and *Salmonella typhi* (MIC, 50 μg/mL).

## 1. Introduction

*Zizyphus* is a genus of about 40 species of spiny shrubs and small trees in the family Rhamnaceae, distributed in warm-temperate and subtropical regions throughout the world. *Zizyphus oxyphylla* Edgew is a small or medium sized tree growing in northern areas of Pakistan. It is used in these areas as a folk medicinal remedy in the treatment of inflammatory conditions, pain, especially of rheumatic origin, fever, microbial infections, allergy and diabetes [1]. *Zizyphus oxyphylla* has been shown to possess analgesic and antipyretic activities in rats and mice [1]. The analgesic and antipyretic activities of some other species of the genus *Zizyphus* have also been reported [1,2]. Cyclopeptide alkaloids are a large group of compounds, which are present in a large number of plant families. Some of the representative families are *Asteraceae*, *Celastraceae*, *Euphorbiaceae*, *Menispermaceae*, *Pandaceae*, *Rubiaceae*, *Sterculiaceae* and *Urticaceae* along with *Rhamnaceae*. These compounds may be defined as basic compounds having a structure 10 or 12 member peptide type bridge span that is attached to benzene at 1,3 or 1,4 positions. These alkaloids have been reported to have antibacterial, antifungal and sedative activity [3].

Plant-derived medicines have been a part of traditional healthcare in most parts of the world for thousands of years and there is increasing interest in plants as the source of agents to fight microbial diseases [4]. As part of our continuous studies on *Zizyphus oxyphylla* [2,5–7], to authenticate its traditional usage, in this paper we report the isolation, the structure elucidation and the antibacterial activity of two new and three known cyclopeptide alkaloids from *Z. oxyphylla.*

## 2. Results and Discussion

**Figure 1:**
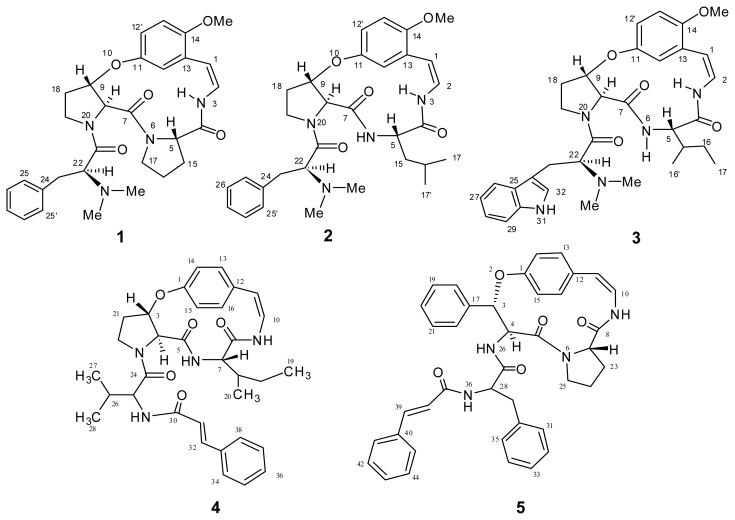
Isolated cyclopeptides (**1**–**5**).

Chloroform fraction of the methanolic extract of Z*. oxyphylla* stem was subjected to multiple chromatographic steps involving column chromatography and preparative TLC. This process yielded two new cyclopeptide alkaloids (**3**,**4**,**5**) and from the dichloromethane fraction of root, two cyclopeptide alkaloids (**1** and **2**) were isolated as white amorphous powders (Figure 1). Structures of the known compounds (**1**–**3**) were elucidated by comparing their analytical data with literature values [6,8–11].

Oxyphylline B (**4**, 13 mg) was obtained as an amorphous solid; its optical rotation [α]D_25_: −76.2 (*c* 0.02, acetone) indicated the presence of chiral centers. UV spectrum showed absorptions at 275 nm, while IR spectrum revealed absorptions at 3300–3400 cm^−1^ (NH), 1656 cm^−1^ (amide), 1508–1624 cm^−1^ (aromatic). EI MS showed M^+^ at *m*/*z* 572, which in combination to ^1^H and ^13^C NMR gives a molecular formula of C_33_H_40_N_4_O_5_. Presence of a peak at *m*/*z* 131 (100%) on mass spectrum implied the presence of cinnamoyl moiety in the molecule.

^1^H NMR spectrum displayed resonances at δ 0.77 d (*J*_20,17_ = 7.5 Hz), 0.78 t (*J*_19,18_ = 7.5 Hz), 0.84 d (*J*_27,26_ = 6.5 Hz) and 0.94 d (*J*_28,26_ = 6.5 Hz), which were assigned to the C-20, C-19, C-27, and C-28 methyl protons respectively. Methine protons resonated at δ 1.97 m, 1.99 m, 4.10 dd (*J*_7,17_ = 9.5, *J*_7,6_ = 4.0 Hz), 4.26 d (*J*_4,3_ = 5.5 Hz), 4.50 t (*J*_25,26/29_ = 8.5 Hz) and 5.45 ddd (*J*_3,21_ = 7.0 Hz, *J*_3,4_ = 5.5 Hz, *J*_3,21′_ = 3.0 Hz) were attributed to the H-26, H-17, H-7, H-4 and H-3, respectively. Olefinic protons at δ 6.10 dd (*J*_10,9_ = 9.5 Hz, *J*_10,11_ = 7.5 Hz) and 6.38 d (*J*_11,10_ = 7.5 Hz) were assigned to the H-10 and H-11, respectively. Coupling constant values indicated that the H-10 and H-11 carbons are *cis* to each other. Similarly, resonances at δ 6.85 d (*J*_31,32_ = 15.5 Hz) and 7.58 d (*J*_32,31_ = 15.5 Hz) were attributed to the H-31 and H-32 protons; their coupling constant values indicated that they are *trans* to each other. Four *ortho* and *meta* coupled protons resonated at δ 7.37 dd (*J*_15,16_ = 8.5 Hz, *J*_16,14_ = 2.5 Hz), 7.06 dd (*J*_14,13_ = 8.5 Hz, *J*_14,15_ = 2.5 Hz), 7.30 dd (*J*_16,15_ = 8.5 Hz, *J*_16,13_ = 2.5 Hz) and 7.18 dd (*J*_13,14_ = 8.5 Hz, *J*_13,16_ = 2.5 Hz), and were assigned to the H-15, H-14, H-16 and H-13, respectively. These two sets of *ortho* coupled protons, confirmed that a *ortho*, *para* substituted benzene ring is present in the molecule. ^1^H-^1^H COSY spectrum indicated the different spin systems in the molecule; H-4 (δ 4.26) exhibited cross peaks with H-3 (δ 5.45) which in turn showed correlations with H_2_-21(δ 1.28 and 2.15). This spin system extended up to H_2_-22 (δ 3.50 and 4.48), revealing the presence of a *β*-substituted proline amino acid unit in the molecule. Similarly, H-7 (δ 3.95) showed cross peaks with H-17, which in turn showed correlation with H_2_-20 and H_2_-18. This spin system extended up to the H_3_-19, thus indicating the presence of an isoleucine amino acid unit as a part of the cyclic ring system. H-10 (δ 6.10) exhibited cross peaks in COSY spectrum with H-9 (δ 7.54) and H-11 (δ 6.38). Similarly, H-25 (δ 4.50) exhibited the cross peaks with H-29 (δ 7.6) and H-26 (δ 1.97); H-26 in turn showed correlations with H-27 (δ 0.84) and H-28 (δ 0.94). This spin system indicated the presence of a valine amino acid unit.

**Figure 2:**
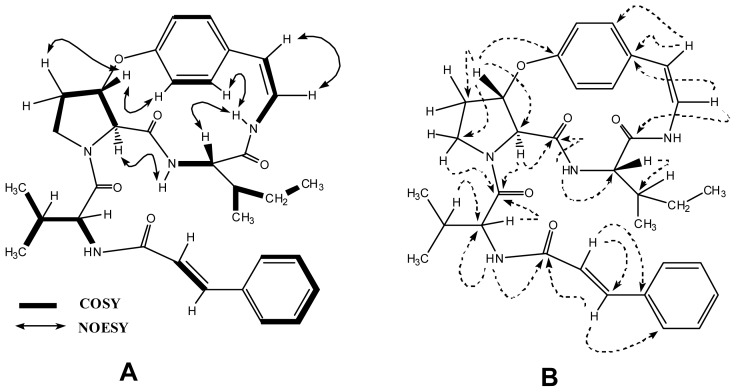
(**A**) Key COSY and NOESY interactions in oxyphylline B (**4**); (**B**) Key HMBC interactions in oxyphylline B (**4**).

^13^C NMR (BB and DEPT) (Table 1) displayed resonances for 33 carbons, including four methyl, three methylene, 19 methine and seven quaternary carbons. The combined use of 1D (^1^H, and ^13^C) and 2D spectra (COSY and HSQC) revealed the different spin systems of the amino acid units such as isoleucine, proline and valine, as well as *p*-oxygenated *Z*-styrylamine and cinnamoyl moiety. For further verification, HMBC experiments were conducted. The HMBC connectivity of H-7 (δ 4.10) to C-5 (δ 172.1), C-8 (δ 168.1), C-17 (δ 37.1) and C-18 (δ 24.8) confirmed the presence of an isoleucine unit as a part of the cyclic ring. Similarly, H-3 (δ 5.80) exhibited the HMBC connectivities with C-21 (δ 32.8), C-4 (δ 65.4), C-1 (δ 158.7), and C-22 (δ 47.2), confirming the presence of a β-substituted proline unit. The correlations of H-10 (δ 6.10) with C-8 (δ 168.1), C-12 (δ 133.4) reconfirmed the presence of a styrylamine moiety. HMBC cross peaks for methine hydrogen H-25 (δ 4.50) to C-24 (δ 172.0), C-30 (δ 166.2), C-26 (δ 31.8) and C-27 (δ 19.4), revealed the fact that the valine amino acid unit has joined the proline amino acid unit and the cinnamoyl moiety. Furthermore, H-31 displayed HMBC correlations with C-30 (δ 166.2) and C-33 (δ 136.3), reconfirming the previous hypothesis. The stereochemistry of the compound was assigned on the basis of coupling constants and NOESY experiments. Key NOESY interactions in compound **4** are shown in Figure 2.

Oxyphylline C (**5**, 19 mg) was obtained as a amorphous solid, its optical rotation [α]D_25_: −49.7 (*c* 0.02, acetone) indicated the presence of chiral centers in the molecule. The IR spectrum exhibited bands corresponding to amine (3375 cm^−1^), amides (1625–1687 cm^−1^) and aromatic 1591 cm^−1^. UV spectrum showed absorption at 283 nm. HRESI MS showed a quasi-molecular ion [M + H]^+^ at *m*/*z* 655.2975, corresponding to the molecular formula C_40_H_38_N_4_O_5_ (calculated C_40_H_38_N_4_O_5_ + H = 655.2920). The ^1^H NMR spectrum (Table 1) displayed signals for double bonds, aromatic protons, methane protons adjacent to heteroatom and methylene protons. Methine protons resonated at δ 3.95 br d (*J*_7,23_ = 7.8 Hz), 4.39 m, 4.80 dd (*J*_4,26_ = 10.2 Hz, *J*_4,3_ = 7.2 Hz), and 5.80 d (*J*_3,4_ = 7.2 Hz) which were ascribed to H-7, H-28, H-4 and H-3 protons, respectively. Olefinic protons at δ 6.55 dd (*J*_10,9_ = 10.0 Hz, *J*_10,11_ = 7.2 Hz) and 6.42 d (*J*_11,10_ = 7.2 Hz), were assigned to H-10 and H-11. Coupling constant values revealed that H-10 and H-11 are *cis* to each other. Similarly, resonances at δ 6.65 d (*J*_38,39_ = 15.0 Hz) and 7.53 d (*J*_39,38_ = 15.0 Hz) were attributed to the H-38 and H-39 protons, and their coupling constant values indicated that they are *trans* to each other. Four *ortho* and *meta* coupled protons resonated at δ 7.0 dd (*J*_15,16_ = 8.4 Hz, *J*_16,14_ = 3.0 Hz), 7.10 dd (*J*_14,13_ = 8.4 Hz, *J*_14,15_ = 3.0 Hz), 7.27 dd (*J*_16,15_ = 8.4 Hz, *J*_16,13_ = 3.0 Hz) and 7.35 dd (*J*_13,14_ = 8.4 Hz, *J*_13,16_ = 3.0 Hz), which were assigned to the H-15, H-14, H-16 and H-13, respectively. These two sets of *ortho*-coupled protons suggested that an *ortho para* substituted benzene ring is present in the molecule. Fifteen other overlapped aromatic protons at the range of δ 7.07–7.71 indicated the presence of additional benzene rings as a side chain in the molecule. ^1^H-^1^H COSY spectrum indicated the different spin systems in the molecule, H-4 (δ 4.80) exhibited cross peaks with H-3 (δ 5.80) and H-26 (δ 7.78). Similarly, H-7 (δ 3.95) showed cross peaks with H_2_-23, which in turn showed correlation with H_2_-24. This spin system extended to the H_2_-25, indicating the presence of a proline ring as a part of the cyclic ring system. H-10 (δ 6.65) exhibited cross peaks in the COSY spectrum with H-9 (δ 7.51) and H-11 (δ 6.42). Similarly, H-28 (δ 4.39) exhibited the cross peaks with H_2_-29 (δ 2.73, 3.29) and H-36 (δ 7.11). ^13^C NMR (BB and DEPT) (Table 1) displayed resonances for 40 carbons, including four methylene, 27 methine and nine quaternary carbons. The combined use of 1D (^1^H, and ^13^C) and 2D spectra (COSY and HSQC) revealed the different spin systems of the amino acid units, such as phenylalanine and proline as well as *p*-oxygenated *Z*-styrylamine and cinnamoyl moieties.

**Figure 3:**
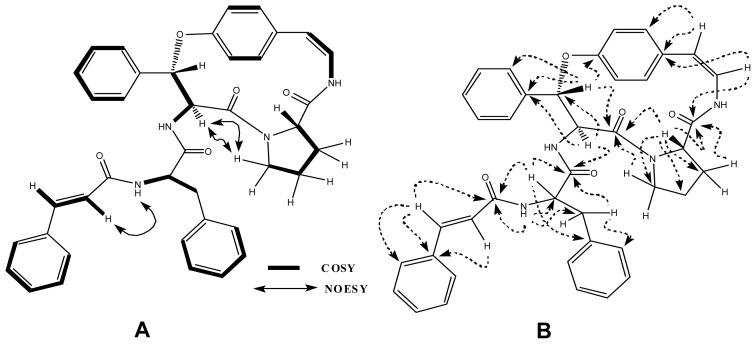
(**A**) Key COSY and NOESY interactions in oxyphylline C (**5**); (**B**) Key HMBC interactions in oxyphylline C (**5**).

An HMBC experiment was used to assemble the different spin systems, functionalities and positions of hetero atoms (Table 1). The HMBC connectivity of H-7 (δ 3.95) to C-5 (δ 172.0), C-8 (δ 167.7), C-23 (δ 25.9), C-24 (δ 25.4) and C-25 (δ 47.9) confirmed the presence of a proline moiety in the cyclic ring. Similarly, H-3 (δ 5.80) exhibited HMBC connectivities with C-5 (δ 172.0), C-4 (δ 57.0), C-1 (δ 156.8), C-17 (δ 139.0), confirming the presence of a β-substituted phenylalanine unit. The correlation of H-10 (δ 6.55) with C-8 (δ 167.7), C-12 (δ 133.0) reconfirmed the presence of a styrylamine moiety. HMBC cross peaks for methine hydrogen (H-28) to C-27 (δ 169.7), C-37 (δ 166.4), C-29 (δ 37.0) and C-30 (δ 138.5), suggested the presence of an α-phenylalanine unit. Furthermore, H-38 displayed HMBC correlations with C-37 (δ 166.4) and C-40 (δ 136.2), indicating that cinnamoyl moiety joined to the α-phenylalanine unit. The NOESY spectrum indicated that the proline unit is part of a cyclic ring, through correlation between H-4 (δ 4.80) and H_2_-25 (δ 3.07 and 3.28). The NOESY correlation between H-38 (δ 6.65) and NH-36 (δ 7.11) reconfirmed the attachment of cinnamoyl moiety with α-phenylalanine unit. Key NOESY correlations are shown in Figure 3. The β-substituted phenylalanine moiety was identified as an erythro moiety on the basis of coupling constant (*J*_3,4_ = 7.2 Hz) [12].

Isolated cyclopeptides showed weak activity against tested bacterial pathogens (Table 2). Oxyphylline B (**4**) showed comparatively better antibacterial activities against *E. coli* (MIC, 5 μg/mL). It also revealed weak antimicrobial activities against *S. aureus* (MIC, 25 μg/mL) *P. aeruginosa* (MIC, 50 μg/mL) and *S. typhi* (MIC, 50 μg/mL). Oxyphylline D (**1**), nummularin-C (**2**), nummularin-R (**3**) and oxyphylline C (**5**) exhibited low antibacterial activities. A possible optimization via derivatization of oxyphylline B (**4**) can be required to study its structure-relationship. Moreover, the investigation of the bacterial molecular target(s) can facilitate the knowledge of its molecular mechanism of action.

## 3. Experimental Section

### 3.1. General Procedures

UV spectra were recorded in MeOH on a Shimadzu UV-240 spectrometer. IR spectra were obtained on a JASCO A-302 spectrophotometer as KBr discs. ^1^H and ^13^C NMR (500 MHz and 125 MHz) were recorded in C_3_D_6_O on a Bruker Av 500 NMR instrument, with TMS as internal standard. HRESI-MS spectra were recorded on QSTAR XL LC MS MS applied bio systems spectrometer. Column chromatography was conducted on silica gel (Kiesegel 60; 70–230 mesh) and TLC was carried out by using pre-coated silica-gel F254 aluminum sheets (0.25 mm thickness). Compounds were detected by spraying with Dragendorff reagent.

### 3.2. Plant Material Extraction and Isolation of Compounds

**Figure 4:**
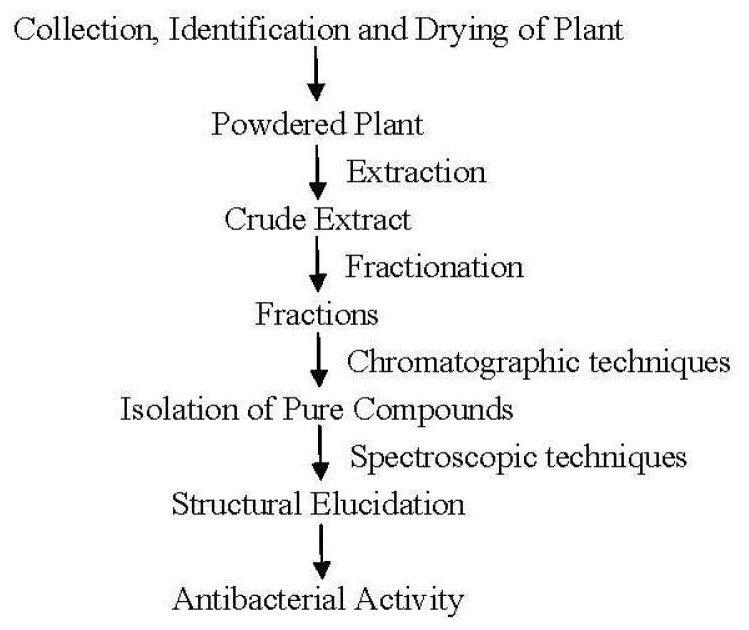
Schematic diagram indicating study design.

The air-dried root powder of *Zizyphus oxyphylla* (8 kg) was macerated in methanol (3 × 7 days × 20 L). After removal of the solvent under vacuum at 35 °C–40 °C, the crude extract (372 g) was obtained. This extract was partitioned between water and dichloromethane. Dichloromethane extract (2.5 g) was subjected to column chromatography over silica gel with hexane/acetone/diethyl amine (75:25:0.1, 10 L) mixture to afford eight fractions (A–H). Compounds **1** (10.2 mg) and **2** (8.7 mg) were obtained from fractions D (41.3 mg) and E (53.4 mg) by preparative TLC (hexane/acetone/diethyl amine, 15:10:1). The air-dried stem powder of *Zizyphus oxyphylla* (8 kg) was macerated in methanol (3 × 7 days × 20 L). Solvent was removed under reduced pressure at 35 °C–40 °C to give the crude extract (375 g), which was partitioned between water and chloroform. Chloroform extract (11.0 g) was purified by column chromatography over silica gel with hexane/acetone/diethyl amine mixture (75:25:0.1, 10 L) to give eight fractions (A–H). Fraction C (202 mg) was subjected to preparative TLC (hexane/acetone/diethyl amine, 15:10:1) to obtain compound **3** (6.1 mg). Fraction D (1.89 g) was subjected to column chromatography over silica gel using chloroform/methanol (89:11) gradient. Compounds **4** (Oxyphylline B, 13 mg) and **5** (Oxyphylline C, 19 mg) were recovered. (See Figure 4.)

### 3.3. Antibacterial Activity

All compounds were screened for antibacterial activity against *Escherchia coli* (ATCC 25922), *Bacillus subtilis* (ATCC 6633), *Shigella flexeneri* (clinical isolate), *Staphylococcus aureus* (ATCC 25923), *Pseudomonas aeruginosa* (ATCC 27853), and *Salmonella typhi* (ATCC 19430), according to the method as reported previously [13–15]. The disc diffusion technique was used to determine the antibacterial activity of the test compounds. The compounds were dissolved in DMSO to obtain a 10 mg/mL solution. A known volume (10 mL) of the solution was applied on to the sterilized filter paper discs with the help of a micropipette. The discs were dried at room temperature overnight and stored in sterile dry containers. Discs, soaked with 10 mL of DMSO, and air dried at room temperature, were used as negative control. Bacterial cultures were grown in a nutrient broth medium at 37 °C overnight and spread on to solidified nutrient agar medium in Petri plates using sterilized cotton swabs in a standard microbiological working environment. Test and control discs were then applied to the solidified medium surface with the help of sterilized forceps. The plates were incubated at 37 °C for 12–15 h. The results were recorded by measuring the zone of inhibition in mm against each compound. Impenem was used as standard drug. MIC was calculated as previously reported [13–15]. Compounds were briefly dissolved in DMSO and serially diluted with sterile water in microplates in a laminar flow cabinet. The same volume of an actively rowing culture of the test bacteria was added to the different wells and cultures were grown overnight in 100% relative humidity at 37 °C. The next morning, tetrazolium violet was added to all the wells. Growth was indicated by a violet color of the culture. The lowest concentration of the test solution that led to an inhibition of growth was taken as the MIC. The negative control DMSO had no influence on the growth at the highest concentration used. Impenem was used as controls for comparison (See Table 1).

## 4. Conclusions

Overuse of antibiotics has become the major factor for the emergence and dissemination of multi-drug resistant strains of several groups of microorganisms. Thus, there is urgent need to develop new antimicrobial agents that are very effective with minimal unwanted side effects. As higher plants have proved to be a source of lead compounds against various infectious diseases, therefore, five cyclopeptide alkaloids, including 2 new cyclopeptide alkaloids isolated from *Z. oxyphylla*, were screened for antibacterial activity. Results indicated weak antimicrobial potential of all isolated cyclopeptide alkaloids.

## Figures and Tables

**Table 1:** ^13^H-NMR (500 MHz) and ^13^C NMR (125 MHz) data for Oxyphylline B (**4**) and Oxyphylline C (**5**).

Position	Oxyphylline B (4)		Oxyphylline C (5)	
	
	δH (J in Hz)	δC, Multiplicity	δH (J in Hz)	δC, Multiplicity
1	-	158.7, C	-	156.8, C
3	5.45 ddd (7.0, 5.5, 3.0)	85.3, CH	5.80 d (*J*_3,4_ = 7.2 Hz)	83.8, CH
4	4.26 d (5.5)	65.4, CH	4.80 dd (*J*_4,26_ = 10.2, *J*_4,3_ = 7.2 Hz)	57.0, CH
5	-	172.1, C		172.0, CO
6	6.90 d (4.0), NH	-		
7	4.10 dd (9.5, 4.0)	59.2,CH	3.95 br d (*J*_7,23_ = 7.8 Hz)	59.8, CH
8	-	168.1, C	_	167.7, CO
9	7.54 d (9.5)	-, NH	7.51 d (*J*_9,10_ = 10.0 Hz)	_, NH
10	6.10 dd (9.5, 7.5)	126.6, CH	6.65 dd (*J*_10,9_ = 10.0 Hz, *J*_10,11_ = 7.2 Hz)	126.7, CH
11	6.38 d (7.5)	117.3, CH	6.42 d (*J*_11,10_ = 7.2 Hz)	116.7, CH
12	-	133.4, C	-	133.0, C
13	7.18 dd (8.5, 2.5)	123.6, CH	7.35 dd (*J*_13,14_ = 8.4 Hz, *J*_13,16_ = 3.0 Hz)	123.2, CH
14	7.06 dd (8.5, 2.5)	131.5, CH	7.1 dd (*J*_14,13_ = 8.4 Hz, *J*_14,15_ = 3.0 Hz)	131.4, CH
15	7.37 (8.5,2.5)	130.3, CH	7.0 dd (*J*_15,16_ = 8.4 Hz, *J*_15,14_ = 3.0 Hz)	132.5, CH
16	7.30 dd (8.5, 2.5)	122.7, CH	7.27 dd (*J*_16,15_ = 8.4 Hz, *J*_16,13_ = 3.0 Hz)	123.9, CH
17	1.99 m	37.1, CH	_	139.0, C
18	1.11 m, 1.59 m	24.8, CH_2_	7.71 br. d (*J*_18,19_ = 7.2 Hz)	129.0, CH
19	0.78 t (7.5)	16.2, CH_3_	7.15 br t (7.2)	128.6, CH
20	0.77 d (7.5)	12.3, CH_3_	7.29 br t (7.2)	128.7, CH
21	1.28 m, 2.15 m	32.8, CH_2_	7.15 br t (7.2)	128.6, CH
22	3.50 m, 4.48 m	47.2, CH_2_	7.71 br. d (*J*_18,19_ = 7.2 Hz)	129.0, CH
23	-		1.51 m, 2.11 m	25.9, CH_2_
24	-	172.0, C	1.35 m, 1.74 m	25.1, CH_2_
25	4.50 t (8.5)	56.6, CH	3.07 t (8.4), 3.28 m	47.4, CH_2_
26	1.97 m	31.8, CH	7.78 d (*J*_26,4_ = 10.2 Hz)	NH
27	0.84 d (6.5)	19.4, CH_3_	_	169.7, CO
28	0.94 d (6.5)	19.1, CH_3_	4.39 m	56.1,CH
29	7.6 d (8.5)	-, NH	2.73 dd (11.4,10.8), 3.29 dd ( 11.4,3.6)	37.0, CH_2_
30	-	166.2, C	-	138.5, C
31	6.85 d (15.5)	122.3, CH	7.12 br d (7.2)	129.8, CH
32	7.58 d (15.5)	141.0, CH	7.20 br t (7.2)	129.3, CH
33	-	136.3, C	7.15 br t (7.2)	130.7, CH
34	7.39 br d (7.5)	129.7, CH	7.20 br t (7.2)	129.3,CH
35	7.57 br t (7.5)	128.5, CH	7.12 br d (7.2)	129.8,CH
36	7.07 br t (7.5)	132.6, CH	7.11 d (7.5)	_, NH
37	7.39 br d (7.5)	129.7, CH	-	166.4, CO
38	7.57 br t (7.5)	128.5, CH	6.65 d (15.0)	120.0, CH
39		7.53 d (15.0)		142.7, CH
40				136.2, C
41,45		7.10 br d (7.2)		129.0, CH
42,44		7.20 br t (7.2)		128 .6, CH
43		7.07 br t (7.2)		129.8, CH

**Table 2:** Antibacterial activity of the isolated compounds. Data are the Minimum Inhibitory Concentration (MIC, μg/mL).

	MIC (μg/mL)					
	
Compound	*Escherichia coli*	*Bacillus subtilis*	*Shigella flexeneri*	*Staphylococcus aureus*	*Pseudomonas aeruginosa*	*Salmonella typhi*
1	10	25	50	25	50	25
2	10	25	100	25	100	10
3	25	10	100	50	100	50
4	5	10	25	25	50	10
5	10	50	50	10	50	50
Standard	0.0002	0.0005	0.0003	0.0009	0.0021	0.0014
